# Adaptation and Validation of Standardized Aphasia Tests in Different Languages: Lessons from the Boston Diagnostic Aphasia Examination–Short Form in Greek

**DOI:** 10.3233/ben-2009-0256

**Published:** 2010-06-29

**Authors:** Kyrana Tsapkini, Christina Helen Vlahou, Costantin Potagas

**Affiliations:** ^1^Department of NeurologyJohns Hopkins Medical InstitutionsBaltimoreMDUSA; ^2^Department of PsychologyAristotle University of ThessalonikiThessalonikiGreece; ^3^Department of NeurologyUniversity of AthensEginition HospitalAthensGreece

**Keywords:** Aphasia testing, BDAE, Greek, normative data, educational effects, cross-cultural neuropsychology

## Abstract

The aim of the current study was to adapt the Boston Diagnostic Aphasia Examination – Short Form (BDAE-SF) [1] to the Greek language and culture, determine the influence of demographic variables on performance and in particular the effects of age and education, develop normative data, and examine the discriminative validity of the test for acute stroke patients. A sample of 129 community healthy adults participated in the study (66 women), covering a broad range of ages and education levels so as to maximize representation of the Greek population and be able to examine the effects of age and education in language performance. Regression models showed that, overall, younger and more educated individuals presented higher performance on several subtests. Normative data for the Greek population are presented in percentile tables. Neurological patients' performance was compared to that of the neurologically intact population using Wilcoxon's rank sum test and for the most part was found to be significantly inferior, indicating good discriminant validity of the test. Qualitative errors of patients diagnosed with aphasia on the test are presented, and limitations and generalizable strengths of this adaptation are discussed.

## 1. Introduction

The Boston Diagnostic Aphasia Examination (BDAE -3 [1,2]) is extensively used in clinical evaluations for the measurement of aphasic patients' performance in all aspects of language functions, identifying the specific language deficits and the exact profile of differential aphasic syndromes. Further, this test is widely used in research protocols. Initially developed in English, efforts have also been made to adapt this test and create norms for non-English populations [3–6]. A previous study has also presented normative data on the Boston Naming Test, which, in its extended form, comprises a subtest of the BDAE [7]. There has also been a preliminary attempt to provide some normative data of the previous full version of BDAE-2 [8] without, however, providing any data from aphasic patients' performance that would assess the discriminant validity of the full-test adaptation.

A short form of its third edition (BDAE-SF) was designed as a brief assessment tool for several language aspects in the 3rd version of the BDAE [1,2]. Given the need for screening tools that could be administered under the time limits frequently imposed in medical settings and determine the need for further referral to a

ISSN 0953-4180/09,10/$27.50 © 2009/2010 – IOS Press and the authors. All rights reserved neurolinguist, speech pathologist, neurologist, or other health clinicians, we decided to adapt the *short form* of the original test to the Greek language and culture, mainly for the use of clinicians.

The importance of avoiding direct translation of items, but rather modifying existing tests so that they are culturally relevant and appropriate to use in each different cultural context has been repeatedly stressed [9], and progressively more and more efforts in Greece focus on this endeavor [10]. In language tests, the issue of cultural adaptation is critical, as the cognitive ability of language is impacted by the particular characteristics and linguistic properties of the individual's native tongue as has been particularly shown to be the case in Greek aphasia [11–13]. It is thus important to avoid pitfalls such as concepts being misinterpreted in the process, or use of test items that are not culturally pertinent for language assessment.

The BDAE-SF includes five functional subsections: (1) conversational and expository speech such as simple social responses, free conversation, and picture description; (2) auditory comprehension including word comprehension, commands, and complex ideational material; (3) oral expression, such as automatized sequences, single word repetitions, repetitions of sentences, responsive naming, the Boston Naming Test – Short Form (BNT-SF), screening of special categories; (4) reading, including letter and number recognition, picture-word matching, basic oral word reading, oral reading of sentences with comprehension, reading comprehension of sentences and paragraphs; and (5) writing, including mechanics, dictation writing of primer words, regular phonics and common irregular forms, written naming, narrative writing – mechanics, written vocabulary access, syntax, and adequacy of content.

The aims of the current study were to administer each subtest of the Greek version of the BDAE-SF to a Greek sample in order to: (1) determine the influence of demographic characteristics on performance, as scores on language tasks are clearly related to age and education [1]; (2) create a normative database and use minimum normal controls' scores as indicators of the differentiating cutoff between aphasics with mild deficits and normal controls; (3) compare the performance of normal controls to neurological patients in order to determine that test's ability to discriminate between normal functioning and aphasia. We also aimed to discuss the qualitative errors presented by aphasics in the first section of the test assessing conversational and expository speech.

## 2. Method

### 2.1. Participants

Our sample consisted of 129 community healthy adults (66 women) including a broad range of age and education level. Age and education categories were chosen in accord with the existing literature. In particular, we followed the categories in previous adaptations of the BDAE in other languages such as Spanish [4] as well as in previous normative studies of neuropsychological tests in Greek (see [10], for the adaptation of verbal fluency test into Greek). Specifically, our normal sample was divided into three different age groups (younger adults: 18–39 years old ($N = 37$, or 28.7%); middle-aged adults, 40–59 years old ($N = 43$, or 33.3%); older adults, 60–81 years old ($N = 49$, or 38%); $M = 51.4$, $SD = 16.6$) and three different education groups according to the Greek school system (low education group: 1–9 education years, i.e. mandatory schooling ($N = 25$, or 19/4%); middle education group, i.e. Lyceum: 10–12 education years ($N = 53$, or 40.3%); high education group, i.e. college and postgraduate studies: 13–21 education years ($N = 52$, or 40.3%). We conducted a brief screening interview in order to exclude individuals with a history of a neurological or psychiatric diagnosis, closed head injury, or any condition that might indicate cognitive impairment. Genders did not differ significantly in age or education level achieved. All participants reported that Greek was their first and dominant language and the majority reported that right hand was their dominant hand preference.

Participants in the normative sample were recruited from a large metropolitan area in Northern Greece. The criterion for participant selection was the recruitment of a stratified sample representing a range of age and education levels. Participants were approached in the community (sample of convenience) by trained psychology undergraduates and graduate students and offered their participation voluntarily. Students were trained and supervised during the data collection process by the first author (K.T.). The test was administered individually, in a quiet, private setting in the community. Administration instructions and procedures followed closely those of the English version of the test. Neurological patients who were hospitalized and treated for a left hemisphere CVA and clinically diagnosed with aphasia subsequent to the stroke were offered participation in a medical setting and tested at bedside by a trained psychology graduate student supervised by the last author (C.P). Written consent was obtained from all control participants and oral consent from all patients. All data were collected in compliance with the Helsinki declaration.

In addition to the normative sample, a sample of 16 neurological patients who had been previously diagnosed with aphasia secondary to a stroke was recruited for comparison in performance scores. The patients' age ranged from 47 to 87 years old ($M = 65.8$, $SD = 12.5$), and their education level from 4 to 20 years of education.

### 2.2. Procedure

Given the specific characteristics of the Greek language, we adapted the BDAE-SF (3rd Ed.) to the need of an appropriate test for the assessment of aphasic disorders in Greek, and developed a version of the BDAE-SF for the Greek population. Examples of the adjustments made include replacing names of US cities (e.g., New York) with Greek cities of similar proportion (i.e., Athens), or providing as multiple choice options Greek words that follow the rationale of the word selection in the original test (e.g., selection of options for the target word included a word that rhymes, a semantically-related word, and a phonologically related word, all in the Greek language, following the example of the English version of the test). The Greek version of the BDAE-SF includes the same five language functional subsections and subtests as the English one.

## 3. Results

### 3.1. Contribution of age, education and sex in the variation of subtests

We examined the contribution of age, education, and sex in explaining the variation of each language subtest. All results were analyzed using the free statistical software 'R' (http://cran.cnr.berkeley.edu/). Understanding the contribution of each factor is easier if the factors do not interact with each other. For this reason, for each language subset, first we assessed the fit of a linear regression model that included all three factors (age, education, and sex) additively with no interaction, compared to the saturated model with all interactions (3 levels for age $\times$ 3 levels for education $\times$ 2 levels for sex).

For the reading subtest, the additive model for the three explanatory factors was found to be very good (the additional $R^2$ of the saturated model was 0.0%, $F(10, 123) = 0.985$, $p = 0.460$)). The results of the additive model, given in Table 1, show that education explains close to 17% ($p < 0.001$) of the variation in the subtest, and this is due to the people with 1–9 yrs of education scoring lower on average than the others. The other factors were not numerically or statistically significant.

For the auditory comprehension subtest, the additive model for the three explanatory factors had a relatively good fit compared to the saturated model (the additional $R^2$ of the saturated model was 5.0%, $F(10, 123) = 1.763$, $p = 0.075$)). The results of the additive model, given in Table 2, show that education explains 8.3% ($p < 0.001$) of the variation in the subtest, and, as with reading, this is due to the people with 1–9 yrs of education scoring lower on average than the others. The other factors did not explain any variation in the subtest.

For the oral expression subtest, the difference in fit between the additive model and the saturated model was statistically significant but again relatively small (the additional $R^2$ of the saturated model was 5.2%, $F(10, 123) = 1.939$, $p = 0.047$)). For this reason, the results of the additive model (given in Table 3) are still useful, and show that age and education together explain 36.4% of the variation. Specifically, in this additive model: (i) people with only 9 yrs of education perform lower than those with 12 yrs of education, and the latter perform lower than those with higher education (contrasts significant at 0.05); and (ii) people older than 40 yrs performed lower than the others. Figure 1 depicts the sources of these differences in the saturated (full interaction) model. As it is shown, older people with low education performed significantly worse than their age group peers with mid- and high education. Furthermore, education did not differentiate the performance in the middle age group but it did in the younger group, i.e. young people with high education performed significantly better than their peers with mid education and better than any other age and education group.

For the writing subtest, as with oral comprehension, the difference in fit between the additive model and the saturated model was statistically significant but relatively small (the additional $R^2$ of the saturated model was 5.3%, $F(10, 123) = 2.90$, $p = 0.003$)). The results of the additive model (given in Table 4) show that age and education together explain 60.3% of the variation. In the additive model: (i) people with low education perform worse than the others; and (ii) older women

Table 1
Predictors of the reading subtest

| | Overall results | | | Contribution of each factor | | |
|---|---|---|---|---|---|---|
| | $R^2$(1) | F | p | Coef (2) | t(df) | p |
| all factors | 19.6% | 7,23 (5, 123) | < 0.0001 | | | |
| age | 0.0% | 0.74 (2, 125) | 0.48 | | | |
| 18–39 (ref) | | | | – | – | – |
| 40–60 | | | | −0.14 | −1.13 (123) | 0.261 |
| 60+ | | | | −0.004 | −0.29 (123) | 0.771 |
| education | 17.1% | 14.3 (2,125) | < 0.0001 | | | |
| > 12 (ref) | | | | – | – | – |
| 10–12 | | | | 0.05 | 0.45 (123) | 0.654 |
| 1–9 | | | | −0.65 | −4.71 (123) | < 0.0001 |
| gender | 0.0% | 1.68(1,124) | 0.197 | | | |
| male (ref) | | | | – | – | – |
| female | | | | −0.12 | −1.30(123) | 0.197 |

(1) R-squares are relative to not having that factor when the others are present; these R-squares do not add up to the total.
(2) Residual standard deviation: 0.53.

Table 2
Predictors of the auditory comprehension subtest

| | Overall results | | | Contribution of each factor | | |
|---|---|---|---|---|---|---|
| | $R^2$(1) | F | p | Coef(2) | t(df) | p |
| all factors | 8.3% | 3.32 (5, 123) | 0.01 | | | |
| age | 0.0% | 0.93 (2, 125) | 0.40 | | | |
| 18–39 (ref) | | | | – | – | – |
| 40–60 | | | | 0.15 | 0.63 (123) | 0.53 |
| 60+ | | | | 0.32 | 1.35 (123) | 0.18 |
| education | 8.3% | 8.27 (2, 125) | < 0.001 | | | |
| > 12 (ref) | | | | – | – | – |
| 10–12 | | | | −0.18 | −0.90 (123) | 0.37 |
| 1–9 | | | | −1.05 | −4.00 (123) | 0.001 |
| gender | 0.0% | 0.04 (1, 124) | 0.85 | | | |
| male (ref) | | | | – | – | – |
| female | | | | 0.03 | 0.19 | 0.085 |

(1) R-squares are relative to not having that factor when the others are present; these R-squares do not add up to the total.
(2) Residual standard deviation: 1.01.

Table 3
Predictors of the oral expression subtest

| | Overall results | | | | Contribution of each factor | | |
|---|---|---|---|---|---|---|---|
| | $R^2$(1) | F | p | | Coef (2) | t(df) | p |
| all factors | 36.4% | 15.62 (5, 123) | < 0.0001 | | | | |
| age | 4.6% | 5.49 (2, 125) | 0.005 | | | | |
| 18–39 (ref) | | | | | – | – | – |
| 40–60 | | | | | −0.56 | −2.06 (123) | 0.04 |
| 60+ | | | | | −0.92 | −3.31 (123) | 0.001 |
| | | | | contrast | 0.36 | 1.69 (123) | 0.094 |
| education | 19.8% | 20.48 (2,125) | < 0.0001 | | | | |
| > 12 (ref) | | | | | – | – | – |
| 10–12 | | | | | −1.97 | −6.40 (123) | < 0.0001 |
| 1–9 | | | | | −0.60 | −2.56 (123) | 0.012 |
| | | | | contrast | −1.37 | −5.37 (123) | < 0.0001 |
| gender | 0.0% | 1.14(1, 124) | 0.289 | | | | |
| male (ref) | | | | | – | – | – |
| female | | | | | 0.22 | 1.07(123) | 0.289 |

(1) R-squares are relative to not having that factor when the others are present; these R-squares do not add up to the total.
(2) Residual standard deviation: 1.19.

Table 4
Predictors of the writing subtest

| | Overall results | | | Contribution of each factor | | |
|---|---|---|---|---|---|---|
| | $R^2$ (1) | F | p | Coef (2) | t(df) | p |
| all factors | 60.3% | 39.9 (5, 123) | $< 0.0001$ | | | |
| age | 0.0% | 0.54 (2, 125) | 0.585 | | | |
| 18–39 (ref) | | | | – | – | – |
| 40–60 | | | | 0.21 | 0.45 (123) | 0.653 |
| 60+ | | | | −0.24 | −0.50 (123) | 0.617 |
| education | 50.3% | 80.17 (2, 125) | $< 0.0001$ | | | |
| $> 12$ (ref) | | | | – | – | – |
| 10–12 | | | | −0.24 | −0.60 (123) | 0.548 |
| 0–9 | | | | −6.3 | −11.8 (123) | $< 0.0001$ |
| gender | 1.9% | 6.88 (1, 124) | 0.009 | | | |
| male (ref) | | | | – | – | – |
| female | | | | 0.95 | 2.62 (123) | 0.010 |

(1) R-squares are relative to not having that factor when the others are present; these R-squares do not add up to the total.
(2) Residual standard deviation: 2.05.

| Age | Edcation | men | women |
|---|---|---|---|
| 60+ | 1-9yrs | 44 | 44 |
| | 10-12 | 46 | 46 |
| | >12 | 46 | 47 |
| | | | |
| 40-59 | 1-9yrs | 45 | 46 |
| | 10-12 | 46 | 47 |
| | >12 | 46 | 46 |
| | | | |
| 18-39 | 1-9yrs | | |
| | 10-12 | 46 | 47 |
| | >12 | 48 | 47 |

Fig. 1. Interaction between age and education in the oral expression subtest. Numbers in cells are average scores. Cells of common shade have average scores that are not statistically significantly different from each other. The averages (standard errors) for different shades are: 47.5 (0.3) for clear shade; 46.1 (0.1) for light grey; 43.9 (0.3) for dark grey; and black cells are empty.

performed better than older men. Figure 2 depicts the sources of these differences in the saturated (full interaction) model. As it is shown, not only people with lower education performed differently from those with mid- and high-education in all age groups, but men with lower education performed worse than women from their education group as well.

### 3.2. *Normative data and discriminant validity*

Percentiles were calculated after compiling data into four major categories, i.e. auditory comprehension, oral expression, reading and writing, each of which resulted from the summation of subcategory scores. Percentiles and descriptive statistics of normals' performance are presented in Table 5 (for auditory comprehension and oral expression) and Table 6 (for reading and writing).

To test discriminant validity of this test, we compared the performance of normals to that of stroke patients with the same education, age and sex group. Because within these groups, the scores of the subtests were not normally distributed, we used Wilcoxon's rank sum test with a two-sided type I error of 5%. The maximal number of normals within each group was 10, so a minimum of 2 patients is required to have non-zero power with this test. Below, we present the Wilcoxon test results (W, p-value) for the comparisons that had at least 2 patients.

a) Old (60+), low-education (1–9 years), men (n$\{$aphasics$\}$ $= 7$; n$\{$normals$\}$ $= 9$): Auditory comprehension: $W = 56.5$, $p = 0.009$; oral

Table 5
Normative data for auditory comprehension (AC) and oral expression (OE) stratified by age and education

| | Age 18–39 years Education (years) | | | | | | Age 40–59 years Education (years) | | | | | | Age 60–81 years Education (years) | | | | | |
|---|---|---|---|---|---|---|---|---|---|---|---|---|---|---|---|---|---|---|
| | 1–9 ($n = 0$) | | 10–12 ($n = 17$) | | 13–21 ($n = 20$) | | 1–9 ($n = 8$) | | 10–12 ($n = 18$) | | 13–21 ($n = 17$) | | 1–9 ($n = 17$) | | 10–12 ($n = 17$) | | 13–21 ($n = 15$) | |
| %ile | AC | OE | AC | OE | AC | OE | AC | OE | AC | OE | AC | OE | AC | OE | AC | OE | AC | OE |
| 100 | | | 32.0 | 48.0 | 32.0 | 48.0 | 32.0 | 48.0 | 32.0 | 48.0 | 32.0 | 48.0 | 32.0 | 46.0 | 32.0 | 47.0 | 32.0 | 48.0 |
| 90 | – | – | 32.0 | 48.0 | 32.0 | 48.0 | 32.0 | 48.0 | 32.0 | 48.0 | 32.0 | 48.0 | 32.0 | 45.0 | 32.0 | 47.0 | 32.0 | 48.0 |
| 80 | – | – | 32.0 | 48.0 | 32.0 | 48.0 | 32.0 | 46.4 | 32.0 | 47.2 | 32.0 | 47.4 | 32.0 | 45.0 | 32.0 | 46.4 | 32.0 | 47.0 |
| 70 | – | – | 32.0 | 47.0 | 32.0 | 48.0 | 32.0 | 46.0 | 32.0 | 47.0 | 32.0 | 47.0 | 32.0 | 44.0 | 32.0 | 46.0 | 32.0 | 47.0 |
| 60 | – | – | 32.0 | 47.0 | 32.0 | 48.0 | 32.0 | 45.4 | 32.0 | 46.4 | 32.0 | 47.0 | 31.4 | 44.0 | 32.0 | 46.0 | 32.0 | 47.0 |
| 50 | – | – | 32.0 | 47.0 | 32.0 | 48.0 | 31.8 | 45.0 | 32.0 | 46.0 | 32.0 | 46.0 | 31.0 | 44.0 | 32.0 | 46.0 | 32.0 | 47.0 |
| 40 | – | – | 32.0 | 46.0 | 32.0 | 47.0 | 31.1 | 45.0 | 32.0 | 46.0 | 32.0 | 46.0 | 30.6 | 44.0 | 32.0 | 45.3 | 32.0 | 46.0 |
| 30 | – | – | 32.0 | 46.0 | 32.0 | 47.0 | 30.4 | 44.7 | 32.0 | 46.0 | 31.5 | 46.0 | 30.2 | 43.0 | 32.0 | 45.0 | 32.0 | 46.0 |
| 20 | – | – | 30.5 | 44.6 | 32.0 | 47.0 | 29.8 | 44.0 | 31.4 | 45.0 | 30.6 | 44.6 | 29.0 | 43.0 | 32.0 | 45.0 | 32.0 | 46.0 |
| 10 | – | – | 28.4 | 45.6 | 31.0 | 47.0 | 29.0 | 44.0 | 30.5 | 44.0 | 29.9 | 42.8 | 28.5 | 42.0 | 31.5 | 45.0 | 32.0 | 45.0 |
| M | – | – | 31.2 | 46.3 | 31.9 | 47.5 | 31.1 | 45.4 | 31.7 | 46.2 | 31.5 | 46.1 | 30.1 | 43.9 | 31.1 | 45.8 | 32.0 | 46.5 |
| *SD* | – | – | 2.0 | 1.2 | 0.3 | 0.76 | 1.6 | 1.3 | 0.6 | 1.2 | 0.85 | 1.7 | 1.3 | 1.1 | 0.2 | 0.75 | 0.0 | 0.9 |

Table 6
Normative data for reading (R) and writing (WR) stratified by age and education

| | Age 18–39 years Education (years) | | | | | | Age 40–59 years Education (years) | | | | | | Age 60–81 years Education (years) | | | | | |
|---|---|---|---|---|---|---|---|---|---|---|---|---|---|---|---|---|---|---|
| | 1–9 ($n = 0$) | | 10–12 ($n = 17$) | | 13–21 ($n = 20$) | | 1–9 ($n = 8$) | | 10–12 ($n = 18$) | | 13–21 ($n = 17$) | | 1–9 ($n = 17$) | | 10–12 ($n = 17$) | | 13–21 ($n = 15$) | |
| %ile | R | WR | R | WR | R | WR | R | WR | R | WR | R | WR | R | WR | R | WR | R | WR |
| 100 | – | – | 39.0 | 84.0 | 39.0 | 84.0 | 39.0 | 84.0 | 39.0 | 84.0 | 39.0 | 84.0 | 39.0 | 83.0 | 39.0 | 84.0 | 39.0 | 84.0 |
| 90 | – | – | 39.0 | 84.0 | 39.0 | 84.0 | 39.0 | 84.0 | 39.0 | 84.0 | 39.0 | 84.0 | 39.0 | 81.4 | 39.0 | 84.0 | 39.0 | 84.0 |
| 80 | – | – | 39.0 | 84.0 | 39.0 | 84.0 | 39.0 | 83.2 | 39.0 | 84.0 | 39.0 | 84.0 | 39.0 | 80.4 | 39.0 | 84.0 | 39.0 | 84.0 |
| 70 | – | – | 39.0 | 84.0 | 39.0 | 84.0 | 39.0 | 82.3 | 39.0 | 84.0 | 39.0 | 84.0 | 39.0 | 79.6 | 39.0 | 84.0 | 39.0 | 84.0 |
| 60 | – | – | 39.0 | 84.0 | 39.0 | 84.0 | 38.4 | 80.2 | 39.0 | 84.0 | 39.0 | 84.0 | 39.0 | 79.0 | 39.0 | 84.0 | 39.0 | 84.0 |
| 50 | – | – | 39.0 | 84.0 | 39.0 | 84.0 | 38.0 | 78.5 | 39.0 | 84.0 | 39.0 | 84.0 | 39.0 | 78.0 | 39.0 | 84.0 | 39.0 | 84.0 |
| 40 | – | – | 39.0 | 84.0 | 39.0 | 84.0 | 38.0 | 77.6 | 39.0 | 84.0 | 39.0 | 83.2 | 38.2 | 75.4 | 39.0 | 84.0 | 39.0 | 84.0 |
| 30 | – | – | 39.0 | 83.4 | 39.0 | 84.0 | 37.7 | 76.7 | 39.0 | 84.0 | 39.0 | 83.0 | 38.0 | 74.4 | 39.0 | 83.4 | 39.0 | 84.0 |
| 20 | – | – | 39.0 | 83.0 | 39.0 | 84.0 | 36.8 | 75.8 | 39.0 | 83.0 | 38.0 | 83.0 | 38.0 | 71.4 | 39.0 | 83.0 | 39.0 | 84.0 |
| 10 | – | – | 39.0 | 82.2 | 39.0 | 84.0 | 36.0 | 75.0 | 39.0 | 81.8 | 38.0 | 81.0 | 36.6 | 68.6 | 37.8 | 82.0 | 39.0 | 84.0 |
| M | – | – | 39.0 | 83.5 | 39.0 | 83.9 | 38.0 | 79.3 | 39.0 | 83.5 | 38.8 | 83.4 | 38.4 | 76.4 | 38.8 | 83.6 | 39.0 | 84.0 |
| *SD* | – | – | 0.0 | 1.2 | 0.2 | 0.5 | 1.1 | 3.4 | 0.0 | 1.0 | 0.44 | 1.0 | 1.1 | 4.7 | 0.5 | 0.7 | 0.0 | 0.0 |

| Age | Education | men | women |
|---|---|---|---|
| 60+ | 1-9yrs | 74 | 79 |
| | 10-12 | 83 | 84 |
| | >12 | 84 | 84 |
| | | | |
| 40-59 | 1-9yrs | 79 | 80 |
| | 10-12 | 83 | 84 |
| | >12 | 83 | 84 |
| | | | |
| 18-39 | 1-9yrs | | |
| | 10-12 | 83 | 84 |
| | >12 | 84 | 84 |

Fig. 2. Interaction between age and education and sex and education in the writing subtest. Cells of common shade have average scores that are not statistically significantly different from each other. The averages (standard errors) for different shades are: 83.9 (15.3) for clear shade; 79.0 (17.1) for light grey; 74.4 (22.8) for dark grey; and black cells are empty.

expression: $W = 51 \quad p = 0.005$; reading: (n{aphasics} $= 0$); writing (n{aphasics} $= 2$): $W = 12$, $p = 0.554$

b) Middle aged (40–59), middle education (10–12 years), men (n{aphasics} $= 4$; n{normals} $= 8$): Auditory comprehension: $W = 32$, $p = 0.005$; oral expression: $W = 32 \quad p = 0.005$; reading: (n{aphasics} $= 3$) $W = 24$ $p = 0.003$; writing (n{aphasics} $= 1$)

c) Middle aged (40–59), higher education (13+ years), men (n{aphasics} $= 2$; n{normals} $= 10$): Auditory comprehension: $W = 20$, $p = 0.015$; oral expression: $W = 20 \quad p = 0.015$; reading: (n{aphasics} $= 3$) $W = 10$ $p = 0.035$; writing (n{aphasics} $= 0$).

### 3.3. *Fluency measures*

Qualitative analysis of free conversation (i.e., response to questions regarding occupation, history of events related to the accident, current hospitalization, and general autobiographical information) and picture description (the ‘cookie theft’), both of which are subsections of the conversational and expository speech subtest, revealed different types of errors. We used as fluency measures the ‘cookie theft’ picture description and the spontaneous speech questions. These sections of BDAE-SF were scored from 1–100 according to the instructions (100 depicting fluent speech with complex grammatically correct sentences). Patients scored low in both fluency measures (‘cookie theft’ mean = 40, SD = 20.7; spontaneous speech mean = 58.75, SD = 23.57). In particular, patients presented syntactic errors (e.g. lack or incorrect use of passive voice, lack of anaphoric propositions, pronouns and clitics), elliptic speech (e.g., lack of nouns/verbs, inadequate sentence construction), word finding difficulties, stereotypic phrases and perseverations (e.g. one patient repeated: “working and drinking, working and drinking”), neologisms (e.g., one patient used the word “dapi” instead of the Greek word “doulapi,” meaning cupboard). A translated example of a patient’s speech output when asked to describe the “cookie theft” picture reads as follows: “Mom, how can I say this, in the kitchen, wiping the plate dry, her children, on a stool the boy and his little hand is up, how can I say this, to get the sweets to eat, he ... probably secretly, he extends his hand to give her one, on the water, water, basin, how can I say this, the si ... the sink upside down, and the water is coming out, the water is overflowing.”

## 4. Discussion

In the current study we adapted the Boston Diagnostic Aphasia Examination – Short Form to the Greek language and culture, for use in screening for aphasia and language functioning assessment in acute and sub-acute stroke. We aimed to determine whether demographic variables such as age, gender, and education, would have an effect on performance. Further, we aimed to develop norms for the Greek population, and determine the validity of the test for discriminating between neurological patients and healthy controls.

Our results suggest that amongst the factors we examined, i.e., age, education and gender, it was only education that influenced consistently the scores in all 4 subtests of the battery. In detail, education was the only factor influencing reading and auditory comprehension and it had a main effect in both oral expression and writing. In addition, education interacted with age in both oral expression and writing and in writing only it interacted with gender as well. Amongst education groups, the higher and mid education group (10–12, and 13+, years of education) performed significantly higher than the low education group (1–9 years of education) in all subtests. Furthermore, in the oral expression subtest there was an additional difference between the mid and higher education group as well. Although we are not aware of any studies exploring the influences of demographic variables on the short form of the BDAE, the present findings that emphasize the important role of eduation in language tests is consistent with previous studies on the BDAE [1–4]. Additionally, the differentiation between the 3 eduation levels in the oral expression subtest shows the particular importance of education in oral expression. This finding corresponds very well with our previous finding on the influence of education in oral semantic fluency measures [14]. In the previous study, we also found that education had an incremental effect on semantic fluency, i.e. the lower education group performed worse than the mid-education group, and that group was also worse than the higher education one. Another point we would like to make is that the effect of education becomes more pronounced in old age. In all subtests, within the older population group, those with lower education (1–9 years) performed significantly worse than older people with mid and higher education. Education is, thus, a predictive factor of good language performance, especially in old age.

Many normal controls achieved a full score, as expected. This is a common finding and a common problem in aphasia tests (e.g. [4,15]). This ceiling effect does not invalidate the predictive value (R-square) of the best-fitted linear models we explored. It does imply, though, that an even higher predictive value can be achieved by a two-stage model, predicting first whether or not the individual has a full score, and, if not, then using a linear model. Such a model for the subtests is a subject for future work. Furthermore, the highly predictive effects of education and age of the linear model show that the test is sensitive to language performance across the education and age span.

Most importantly for the purpose of this study, the test had good discriminant validity, as the performance of a small sample of neurological patients already diagnosed with aphasia after a left hemisphere stroke was found to be significantly different than that of the normative sample on most subtests of the test. Writing was the test with the least discriminant validity because most patients had severe motor deficits and could not perform this subtest. To circumvent this generic but quite prevalent problem in acute stroke, we suggest that instead of the actual writing, patients could be asked to spell the words orally. Here, we would also like to make a note that in the cases where a single patient should have been compared to this control group we did not perform Crawford and Garthwaite's [16] very useful method for such comparisons because this method assumes a normal distribution of the control sample, something that was not true in most subtests. (In [16] the authors provide a very useful statistical method that can be applied when one needs to compare a single subject, often a patient in neuropsychology, with a modestly sized matched control sample.) In general, this short version adapted to the Greek culture and language seems to be appropriate for use with stroke patients.

A limitation of this test is that is does not allow for detailed assessment of syntactic, morphosyntactic or morphophonological problems as already found for Greek patients with aphasia [11–13]. Assessment of spontaneous speech is only qualitative, and the measure does not assess degree of severity of deficits or broader functional limitations. Furthermore, our analysis is limited to the group of patients available at the time since their profile of aphasia may change with time. Therefore more research is needed to validate the full battery in chronic stroke when plasticity mechanisms are at work and provide more variable profiles. Alternatively, it comprises a sensitive screening tool that is quick to administer in medical settings for the purpose of diagnosing and further referring patients with aphasia.

The adaptation and validation of the BDAE-SF presented in this study serves as a lesson for the issues, decisions and most importantly the challenges that the clinical researcher faces when adapting a standardized aphasia test in a different language and culture. Besides learning about the effects of education in different language functions and documenting the discriminant validity of this test, the present endeavor shows that when adaptations use principles that respect each language and cultural properties the test preserves its discriminative power to detect language attrition.

## Acknowledgements

We would like to thank all the patients and control subjects who participated in this study. We would also like to thank Anna Emanouil, Kristi Passalidou, and Georgia Nasiopoulou, students at the Master's program in Cognitive Psychology of the Department of Psychology at the University of Thessaloniki, who participated in early control data collection and preliminary analysis for research course credit, as well as Eleni Peristeri for patient data collection and analysis. We are grateful to Dr Constantine Frangakis, Professor of Biostatistics at Johns Hopkins University, for his help with the statistical analysis. No conflicts of interest and no sources of financial support exist for this study.
